# Neurosarcoidosis-Induced Hypophysitis Mimicking Pituitary Macroadenoma

**DOI:** 10.7759/cureus.39865

**Published:** 2023-06-02

**Authors:** Taïeb Ach, Wissal Ben Yahia, Imen Halloul, Fatma Sghaier, Amira Atig

**Affiliations:** 1 Endocrinology, University Hospital of Farhat Hached, Sousse, TUN; 2 Internal Medicine, University Hospital of Farhat Hached, Sousse, TUN

**Keywords:** diabetes insipidus, pituitary, pituitary macroadenoma, hypophysitis, neurosarcoidosis

## Abstract

Sarcoidosis is characterized by the presence of noncaseating granulomatous inflammation in the affected organs. Isolated involvement of the hypothalamic-pituitary axis in patients with sarcoidosis is rare. We report a rare case of a female patient in whom hypophysitis, mimicking a pituitary macroadenoma, resulted in pituitary transsphenoidal surgery. A female patient had been complaining of bilateral temporal headaches for over a month. Brain MRI showed a pituitary adenoma of height 16 mm, width 16 mm, and depth 12 mm. Hormonal assay showed central hypothyroidism and elevated level of prolactin. Histological examination revealed granulomatous hypophysitis. A specific search for Mycobacterium tuberculosis was negative on the pituitary tissue. After the exclusion of differential diagnoses, the combination of clinical, laboratory and radiological tests led to the diagnosis of neurosarcoidosis. This report presents an uncommon case of a pituitary localization of neurosarcoidosis mimicking a macroadenoma. Understanding the different MRI aspects of neurosarcoidosis is essential to avoid interpretive blunders that could result in an incorrect diagnosis.

## Introduction

Hypophysitis is a rare condition characterized by infiltration and possible destruction of the pituitary gland. The incidence is not exactly known but approaches one case per 9 million people per year [1]. Hypophysitis can be classified as primary or secondary. It may be caused, in the latter, by systemic inflammatory disorders [1].

Histologically, hypophysitis is further divided into three pathological subtypes: xanthomatous, lymphocytic, and granulomatous. Granulomatous hypophysitis, such as sarcoidosis, is likely to be the second most common subtype and features widely distributed multinucleated giant cells, histiocytes, some forming granulomas, and variable amounts of lymphocytic infiltration and fibrosis [2].

Sarcoidosis is a multisystem disorder of unknown aetiology, belonging to the spectrum of granulomatosis. Involvement of the central nervous system is a well-known manifestation of the disease occurring in approximately 5% of patients and including some rare cases of hypophysitis [3].

The clinical spectrum of hypophysitis usually includes headache, visual symptoms, and anterior pituitary insufficiencies depending on the extent of inflammation. Enhanced magnetic resonance imaging (MRI) mostly reveals sellar and pituitary stalk lesions, with iso or hypointense signals on T1- and T2-weighted images, a picture that may mimic pituitary adenomas (PA) [4]. It is challenging to diagnose hypophysitis, especially when it mimics other common sellar lesions due to the lack of specific clinical signs. Sarcoidosis-induced hypophysitis (SIH) is a rare sellar entity. Clinically and radiologically, it can be easily misdiagnosed as a pituitary adenoma leading to unjustified pituitary surgery.

Herein, we present a rare case of a female patient diagnosed initially on an MRI secondary to chronic headaches and visual impairment as pituitary macroadenoma, which resulted in a pituitary transsphenoidal surgery rectifying the diagnosis as SIH.

## Case presentation

A 49-year-old woman had been complaining of a bilateral temporal headache for over a month. As for her medical history, she was treated 20 years ago for tuberculous lymphadenitis and had a metabolic syndrome for four years. She started her menarche at the age of 12 and used to have regular menstrual cycles. She had her menopause at the age of 42.

The pain was essentially retro-orbital and was slightly alleviated by analgesics. The headache was associated with inflammatory arthralgia and occasionally with pain and redness in her right eye. The patient denied a history of trauma or seizures; she also reported recent visual blurriness. Physical examination found an alert and conscious patient. Pulmonary auscultation and skin examination were normal. A complete neurologic examination was normal, including sensorium, cranial nerves, motor, sensory, cerebellar and reflexes.

Ophthalmological examination confirmed the presence of right panuveitis with multiple choroidal granulomas.

**Figure 1:**
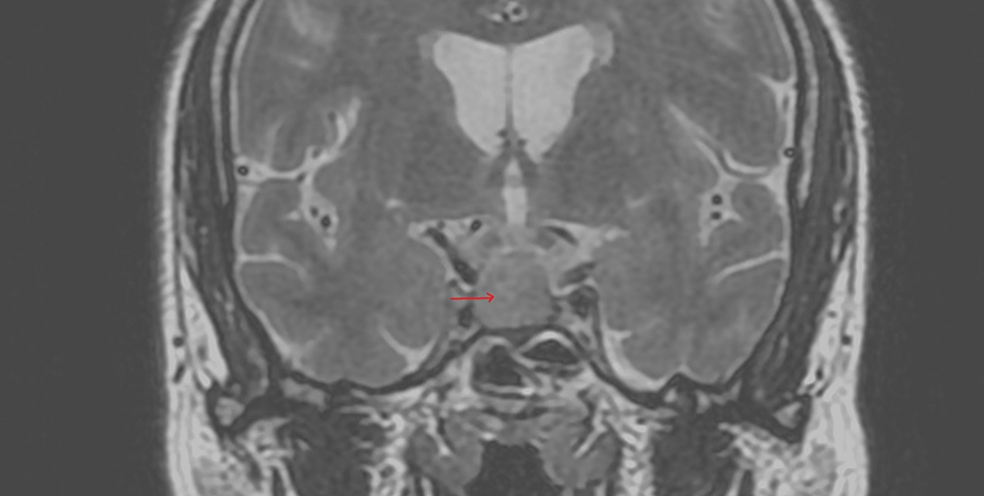
Pituitary MRI with gadolinium contrast; coronal T2W image showing a pituitary macroadenoma of 16x16x12 mm diameter

**Figure 2:**
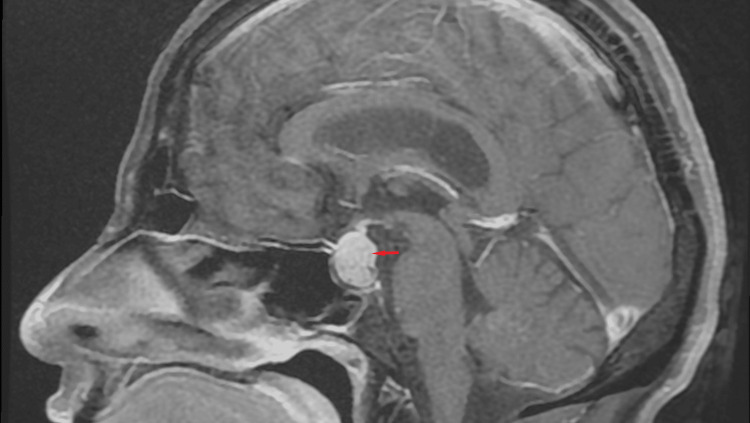
Pituitary sagittal T1W after gadolinium injection MRI image showing a pituitary macroadenoma of 16x16x12 mm diameter with parasellar extension and without compression of the optic chiasm

Brain MRI showed a pituitary adenoma of 16x16x12 mm diameter with parasellar extension and without compression of the optic chiasm. The mass was hypointense on T1- and hyper-intense on T2-weighted images and homogeneously enhanced after the administration of Gadolinium (Figures 1, 2).

A hormonal assay of the pituitary gland at 8 AM showed central hypothyroidism and an elevated level of prolactin. Other pituitary hormones, including morning cortisol, were within normal levels (Table 1).

**Table 1:** Main biological and hormonal assessment FT4: free thyroxine, FSH: follicle-stimulating hormone, LH: luteinizing hormone

Samples	Results	Normal value
Calcium (mmol/L)	2.45	2.2 – 2.6
Calciuria (mmol/24h)	12.3	2.5 - 6
Angiotensin-converting enzyme (ng/mL)	70.2	<5
Sedimentation rate at H1 (mm)	24 mm	0-10
FT4 (pg/L)	6	7-19
FSH (UI/L)	12	5-15
LH (UI/L)	9	5-15
Estradiol (UI/L)	46	>40
Cortisol (ng/mL)	189	>180
Prolactin (ng/L)	50	<30

The diagnosis retained was a pituitary macroadenoma and secondary hypopituitarism. A transsphenoidal surgery was done without any complications. The patient received hydrocortisone and levothyroxine therapy prior to surgery, which were tapered down to a maintenance dose.

Histological examination revealed a granulomatous hypophysitis (epithelioid and giant cells without caseous necrosis). Wegener’s granulomatosis, syphilis, and histiocytosis X were ruled out on the basis of physical examination and initial biological tests. Tuberculosis and sarcoidosis were the most plausible aetiologies. Laboratory parameters showed an elevated angiotensin-converting enzyme (ACE) and hypercalciuria. A chest and abdominal CT scan revealed hepatomegaly but no lymphadenopathies. Polymerase chain reaction, for the specific detection of Mycobacterium tuberculosis, was negative on the pituitary tissue.

After excluding the differential diagnosis, a combination of clinical, laboratory, and radiographic findings led to the diagnosis of neurosarcoidosis. The patient was initially supplemented with desmopressin for a transient post-surgical diabetes insipidus and the hypothyroidism was corrected with standard replacement therapy.

The patient remained on replacement thyroxin and hydrocortisone therapy. She was subsequently switched to prednisone for its anti-inflammatory action for her sarcoidosis at the dose of 1 mg/kg body weight. On the six-month follow-up, clinical examination showed an improvement in uveitis.

## Discussion

In this case, we reported a rare situation of SIH mimicking pituitary macroadenoma. In fact, the clinical presentation of hypophysitis is variable. Headaches and visual abnormalities, such as visual field defects and decreased acuity, are the most common symptoms of sellar compression [5]. Lateral expansion of the mass into the cavernous sinus could compress cranial nerves III, IV or VI, resulting in ocular misalignment and diplopia [5]. In our case, the patient presented with signs of sellar compression including visual symptoms and a chronic headache. In cases of pituitary compression signs, MRI plays an important role in the diagnosis of primary pituitary inflammation, offering a clear advantage over CT. Among MRI findings, diffuse pituitary enlargement, marked enhancement, and stalk thickening are the best predictors of primary pituitary inflammation that can be distinguished from large adenomas [5].

Some hypophysitis can occur with a typical clinical and radiological presentation, including diabetes insipidus and pituitary enlargement, therefore, easily differentiated from PA.

Some granulomatous hypophysitis may occur as pituitary apoplexy or mimic a pituitary abscess [6,7]. SIH mimicking PA has been rarely reported [8]. Both hypophysitis and non-functioning pituitary adenoma can cause pituitary expansion and variable degrees of hypopituitarism. In hypophysitis, however, the level of pituitary insufficiencies is disproportionate compared to the lesion’s volume [8]. In patients with SIH, hypopituitarism can be observed with a small mass or even a normal pituitary gland while in patients with tumours, such a level of panhypopituitarism is commonly seen with larger masses [3]. Diabetes insipidus rarely accompanies a PA and is due to a compression of the stalk. However, in granulomatous hypophysitis, it is one of the most frequent complications caused by the infiltration of the stalk [9].

In our case, the lack of specific symptoms of hypophysitis induced the medical staff to errors and led to surgery in order to save the visual prognosis. In the presence of a pituitary mass and a pituitary hormonal dysfunction, trans-sphenoidal exploration and histological examination are necessary to reach the final diagnosis. Surgery provided tissue for histological diagnosis and allowed the rapid decompression of the lesion, therefore resolving the headache and visual deficits immediately. Histopathological findings allowed for the diagnosis of granulomatous hypophysitis. With the histology evidence and high erythrocyte sedimentation rate (ESR) and ACE levels, the diagnosis of neurosarcoidosis was retained. The most accurate imaging method for diagnosing neurosarcoidosis is brain MRI. The two most typical neurosarcoidosis findings are the periventricular distribution of lesions and leptomeningeal enhancement, which were missing in our case [4]. Along with endocrine complaints, the pituitary stalk may become thicker.

The originality of our case is that surgery, considered later on as a biopsy, indirectly allowed the diagnosis. Neurosarcoidosis with pituitary involvement is rare and has been characterized only through case series [8,10]. In contrast to other localizations of sarcoidosis (which have a gender predisposition), SIH appears to affect more men than women [3,8]. The most frequent neuro-ophthalmologic symptom in neurosarcoidosis patients is anterior uveitis [8]. As was the case with our patient, anterior uveitis is typically the most common reason for vision affection in sarcoidosis patients [3]. The optic chiasma may directly or indirectly cause SIH's optic neuropathy [10].

There are no guidelines on the therapy objectives for SIH, and no controlled prospective trials have been conducted in this area. Two key objectives of medical care for SIH are the replacement of pituitary hormone deficiency and optical care. The main early treatment for individuals with neurosarcoidosis remains with high doses of glucocorticoids [1].

## Conclusions

Involvement of the nervous system in sarcoidosis can occur alone or in conjunction with systemic inflammation. Due to its non-specific symptoms and clinical similarities to other diseases, isolated neurosarcoidosis may be difficult to diagnose. This case describes a rare instance of neurosarcoidosis that mimics a pituitary macroadenoma. Understanding the range of magnetic resonance imaging (MRI) findings in neurosarcoidosis is essential to avoid interpretive blunders that could result in an incorrect diagnosis and course of treatment.
